# Supplementary material for: The role of FRUITFULL controlling cell cycle during early flower development revealed by time-series snRNA-seq experiments
Source: Genome Biol. 2025 Oct 27;26:370. doi: 10.1186/s13059-025-03831-z (PMC12557869; doi:10.1186/s13059-025-03831-z)
Supplement: Supplementary file 1 — Additional file 1. Fig. S1. Quality control of the snRNA-seq experiment. a) Distribution of the number of reads mapped per nucleus. b) Distribution of the number of detected genes per nucleus. c) Proportion of mapped reads located in the chloroplast genome per nucleus. d) Proportion of mapped reads located in the mitochondrial genome per nucleus. Fig. S2. Reproducibility between snRNA-seq and bulk RNA-seq. The scatterplot shows the high reproducibility of the different snRNA-seq datasets against bulk RNA-seq data collected at the same stage and genotype [28] (A-D). For snRNA-seq, read counts per protein-coding gene were summed across all transcriptomes. For bulk RNA-seq, read counts per protein-coding gene were summed across all biological replicates. Read counts per gene were normalized by the total number of mapped reads and multiplied by 10 [6]. Fig. S3. Expression and coexpression of snRNA-seq marker genes. a) Dotplot showing the average expression and percent of cells expressing particular genes mentioned in the main text to help cluster annotation. b) Heatmap showing the Pearson correlation between the average expression of each cluster (rows) and selected bulk RNA-seq data from [45] (columns). To calculate the correlation only genes identified as top50 marker genes for each cluster were used. Fig. S4. Distribution of snRNA-seq samples across the UMAP plot. The UMAP plot shown in Figure 2a is plotted for each snRNA-seq dataset separately. Points are color-coded as Fig 2a to indicate cluster groups. a) it shows only transcriptomes from the DEX-uninduced sample (S0), b) from the S2 sample, c) from the S4, d) from the S8 sample, and e) from the Col-0 sample. f) Average expression of KRP2 among the transcriptomes of each UMAP cluster. g) Annotation of each cluster depending on its expression correlation with bulk RNA-seq (TraVa, flower tissues). The correlation was calculated between the average gene expression for each cluster and TraVA developmental stage-specif [file 13059_2025_3831_MOESM1_ESM.docx]

**
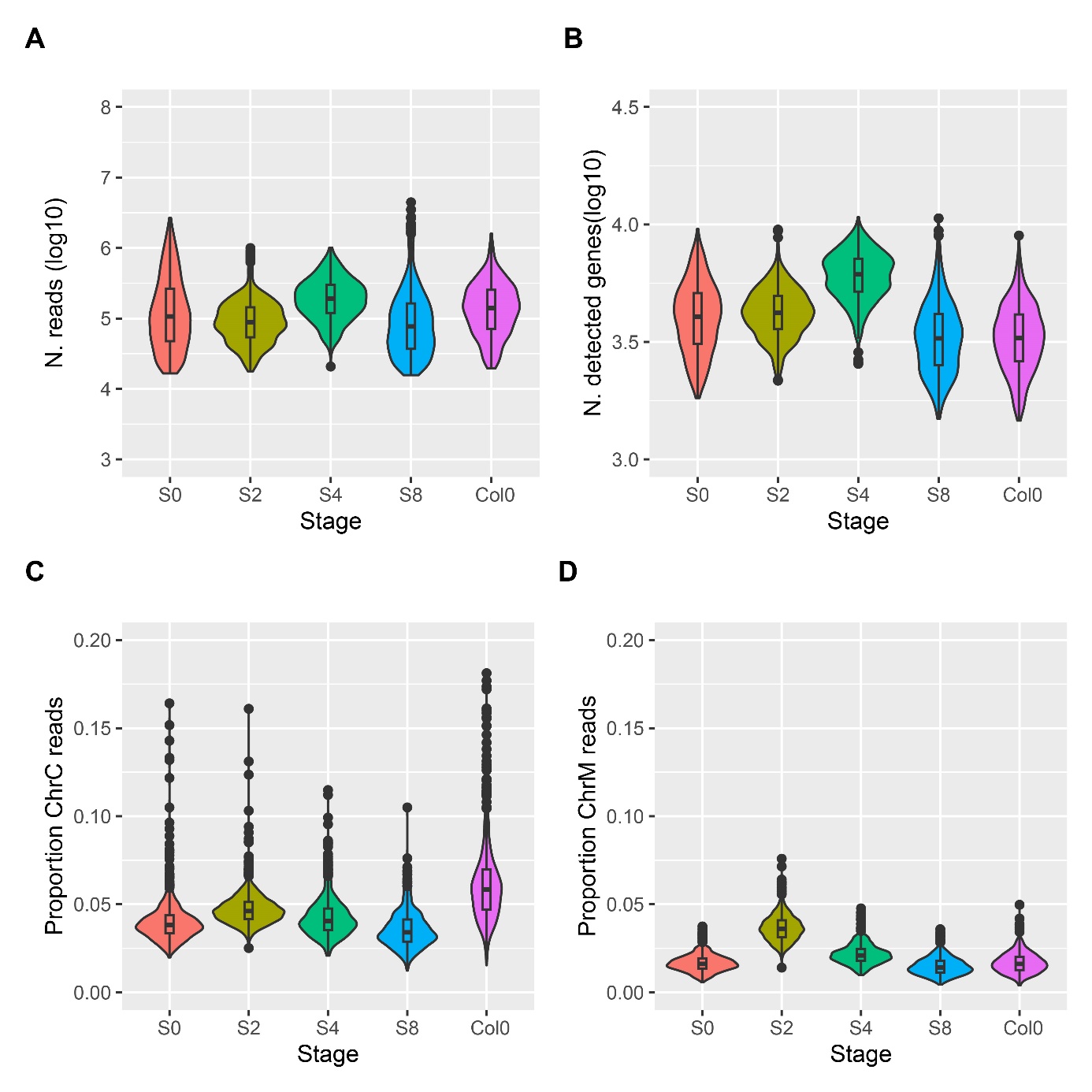
**

**Fig. S1. Quality control of the snRNA-seq experiment. A)** Distribution of the number of reads mapped per nucleus. **B)** Distribution of the number of detected genes per nucleus. **C)** Proportion of mapped reads located in the chloroplast genome per nucleus. **D)** Proportion of mapped reads located in the mitochondrial genome per nucleus.

**
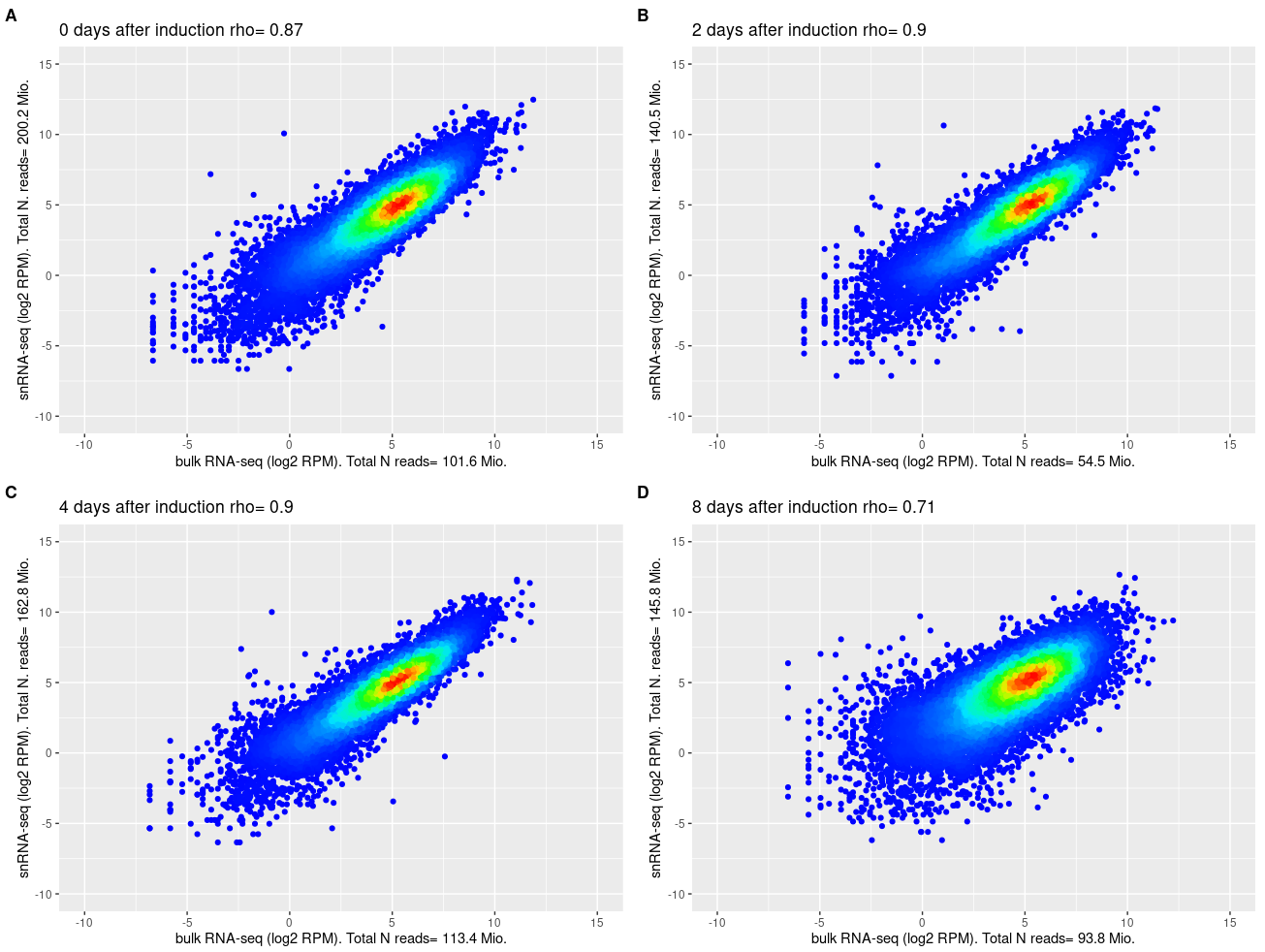
**

**Fig. S2. Reproducibility between snRNA-seq and bulk RNA-seq**. The scatterplot shows the high reproducibility of the different snRNA-seq datasets against bulk RNA-seq data collected at the same stage and genotype (1) (A-D). For snRNA-seq, read counts per protein-coding gene were summed across all transcriptomes. For bulk RNA-seq, read counts per protein-coding gene were summed across all biological replicates. Read counts per gene were normalized by the total number of mapped reads and multiplied by 10^6^.

**
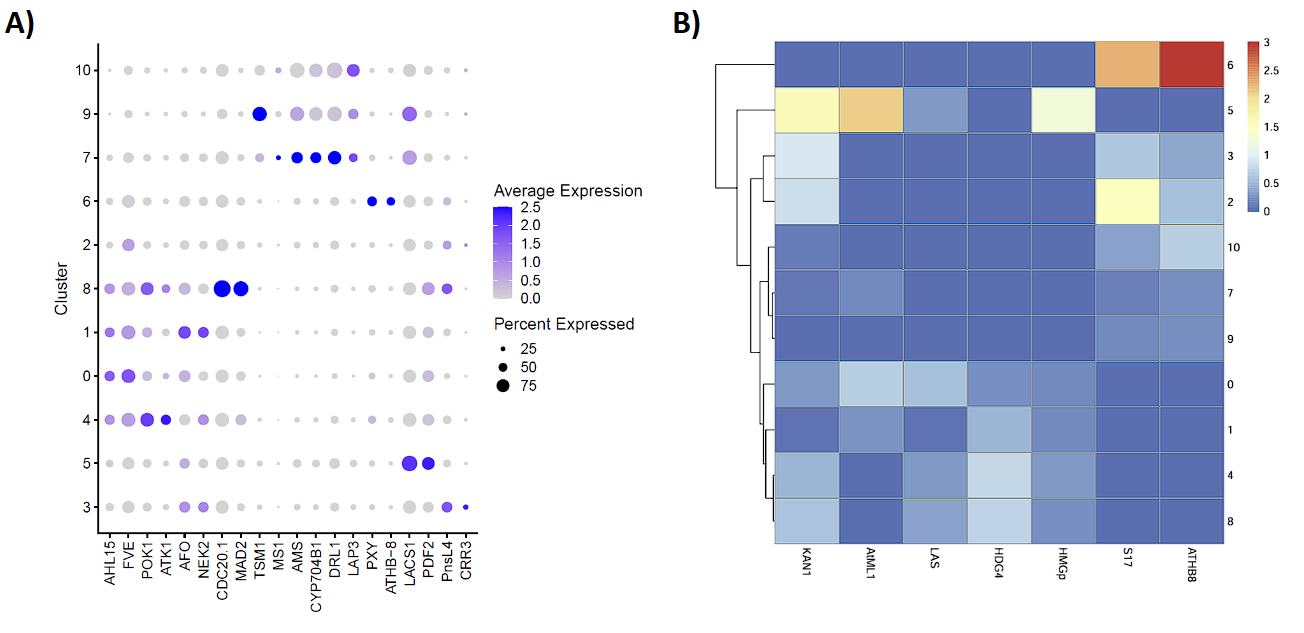
**

**Fig. S3. Expression and coexpression of snRNA-seq marker genes. A)** Dotplot showing the average expression and percent of cells expressing particular genes mentioned in the main text to help cluster annotation. **B)** Heatmap showing the Pearson correlation between the average expression of each cluster (rows) and selected bulk RNA-seq data from (2) (columns). To calculate the correlation only genes identified as top50 marker genes for each cluster were used.

**
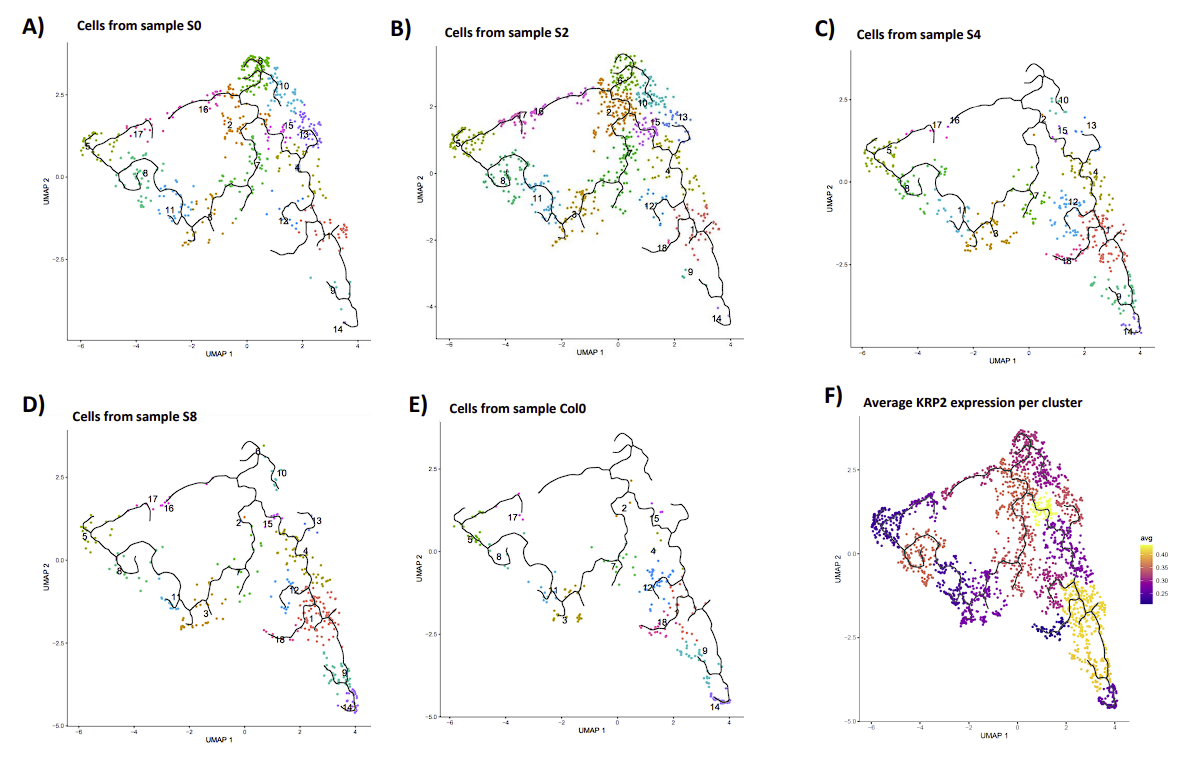
**

**
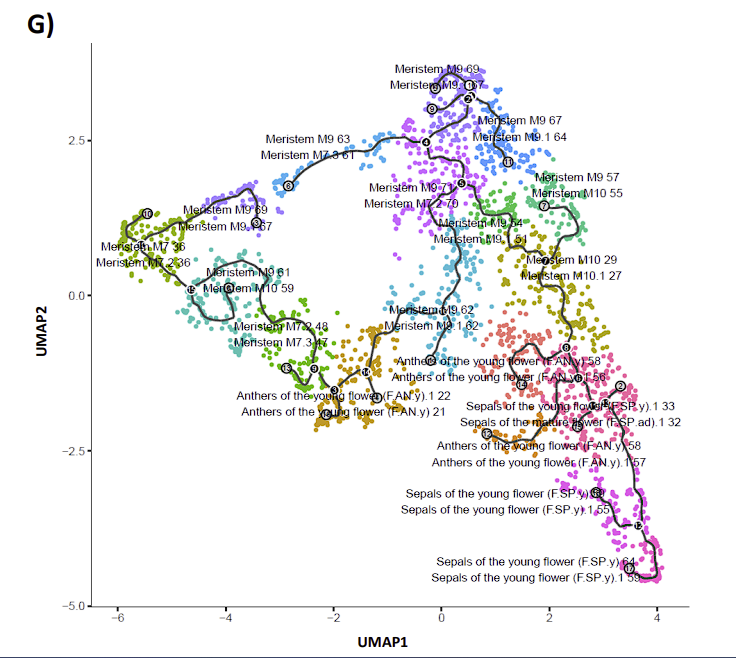
**

**Fig. S4. Distribution of snRNA-seq samples across the UMAP plot**. The UMAP plot shown in Figure 2a is plotted for each snRNA-seq dataset separately. Points are color-coded as Fig 2a to indicate cluster groups. **A**) it shows only transcriptomes from the DEX-uninduced sample (S0), **B**) from the S2 sample, **C**) from the S4, **D**) from the S8 sample, and **E**) from the Col0 sample. **F**) Average expression of KRP2 among the transcriptomes of each UMAP cluster. **G)** Annotation of each cluster depending on its expression correlation with bulk RNA-seq (TraVa, flower tissues). The correlation was calculated between the average gene expression for each cluster and TraVA developmental stage-specific bulk RNA-seq data. The two TraVa datasets with the highest correlation are reported for each cluster. The last 2 digits represent the correlation coefficient *100.

**
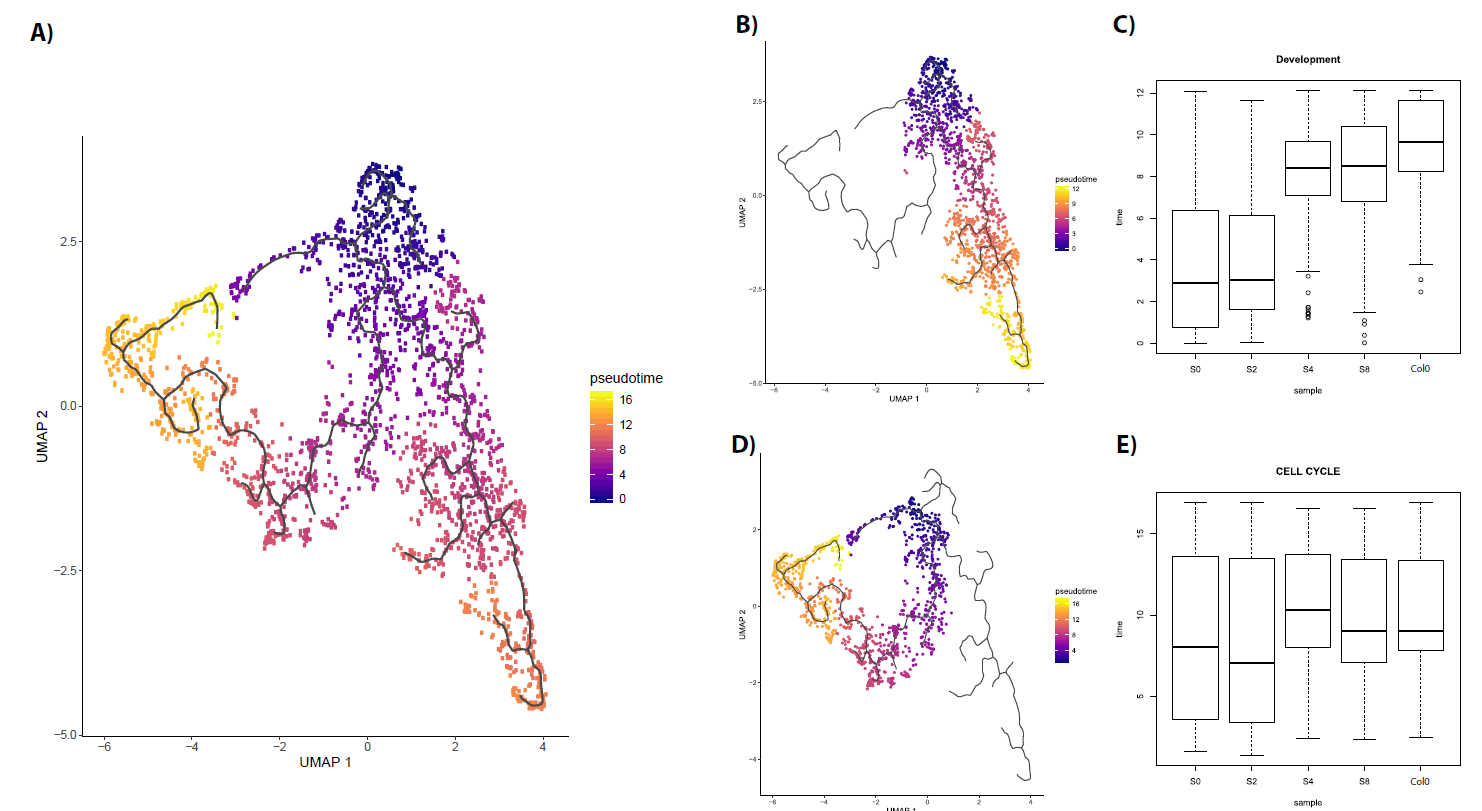
**

**Fig. S5.** **Visualization of pseudo-time estimation in the UMAP plot**. Pseudo-time was estimated for all cells using Monocle3. Cluster 6 was set at pseudo-time 0 as it was the cluster with the highest enrichment in transcriptomes from the snRNA-seq sample S0. **A**) UMAP plot showing the pseudo-time values estimated for each transcriptome. **B**) UMAP plot showing the pseudo-time estimated for each transcriptome of the clusters linked to the development-related trajectory. **C**) Boxplot of pseudo-time values estimated for each transcriptome represented in B) depending from which snRNA-seq sample they belong. The boxplot shows a significant difference in pseudo-time distribution between samples. **D**) UMAP plot showing the pseudo-time values for each transcriptome associated with the cell cycle trajectory. **E**) Boxplot of pseudo-time values estimated for each transcriptome represented in D) depending from which snRNA-seq sample they belong. The boxplot shows no significant differences in pseudo-time distribution between samples.

**
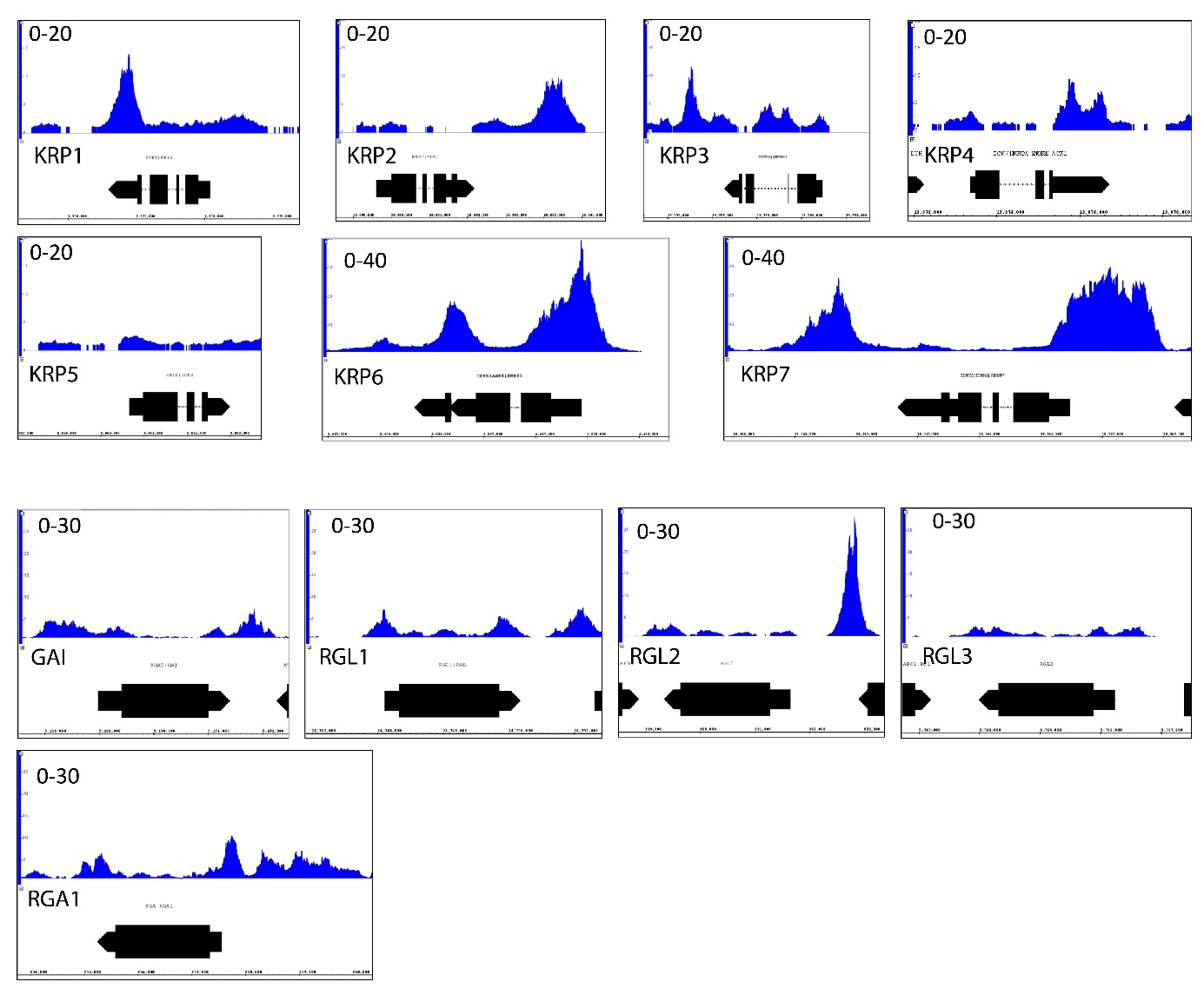
**

**Fig. S6. FUL binding patterns in KRP and DELLA protein-coding genes**. Screenshots of the genomic regions around KRP and DELLA protein-coding genes. The screenshots show FUL DNA binding from ChIP-seq experiments (3). *KRP* genes, except for *KRP5*, have a significant FUL binding peak (FDR<0.05). For *DELLA* genes, only *RGL2* and *RGA1* have a significant FUL binding peak (FDR<0.05). See Supplementary Table S4 for FDR values.

**
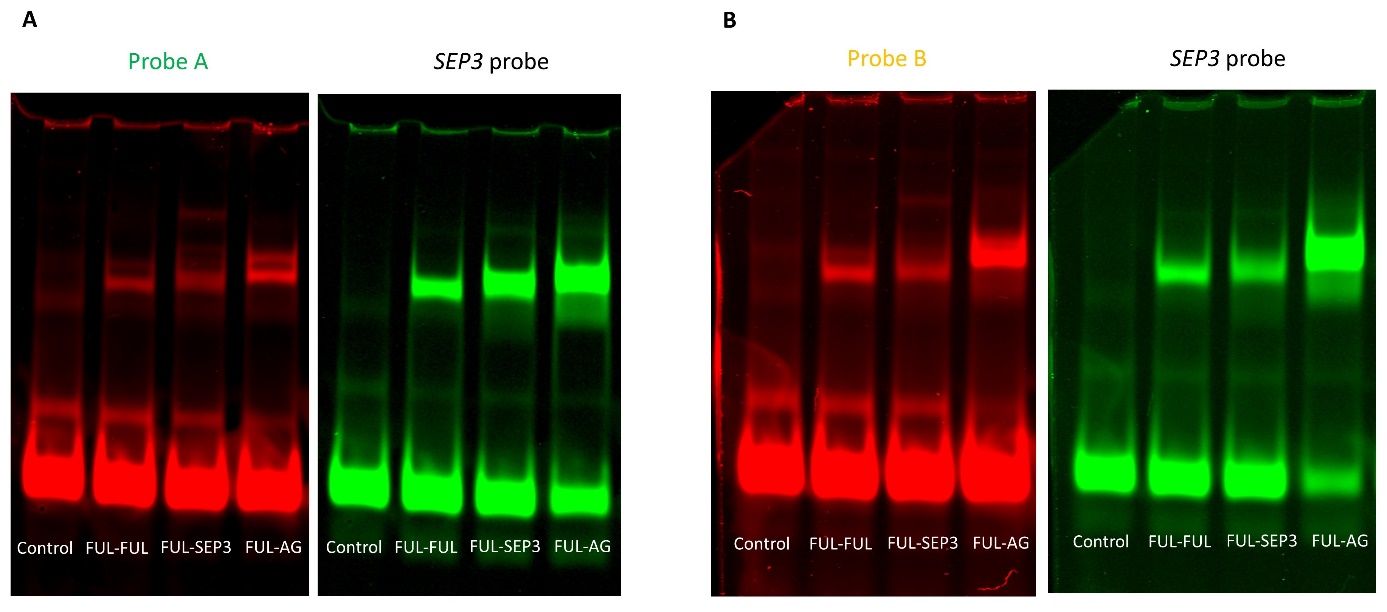
**

**Fig. S7. EMSA showing FUL binding to the *KRP2* downstream genomic region**. EMSA results of *KRP2* probes with different FUL dimers. Probe A and B represented the CArG-box A (A) and B (B) sequences located in the *KRP2* downstream region within the FUL binding peak (see Figure 3a). As positive control, a probe called “*SEP3* probe” representing a DNA fragment from *SEPALLATA3* promoter region (a shorter version of “SEP3 wt” from(4) was used. The dimer tested were FUL homodimer (FUL) and FUL heterodimers with AGAMOUS (FUL-AG) and SEPALLATA3 (FUL-SEP3).

**
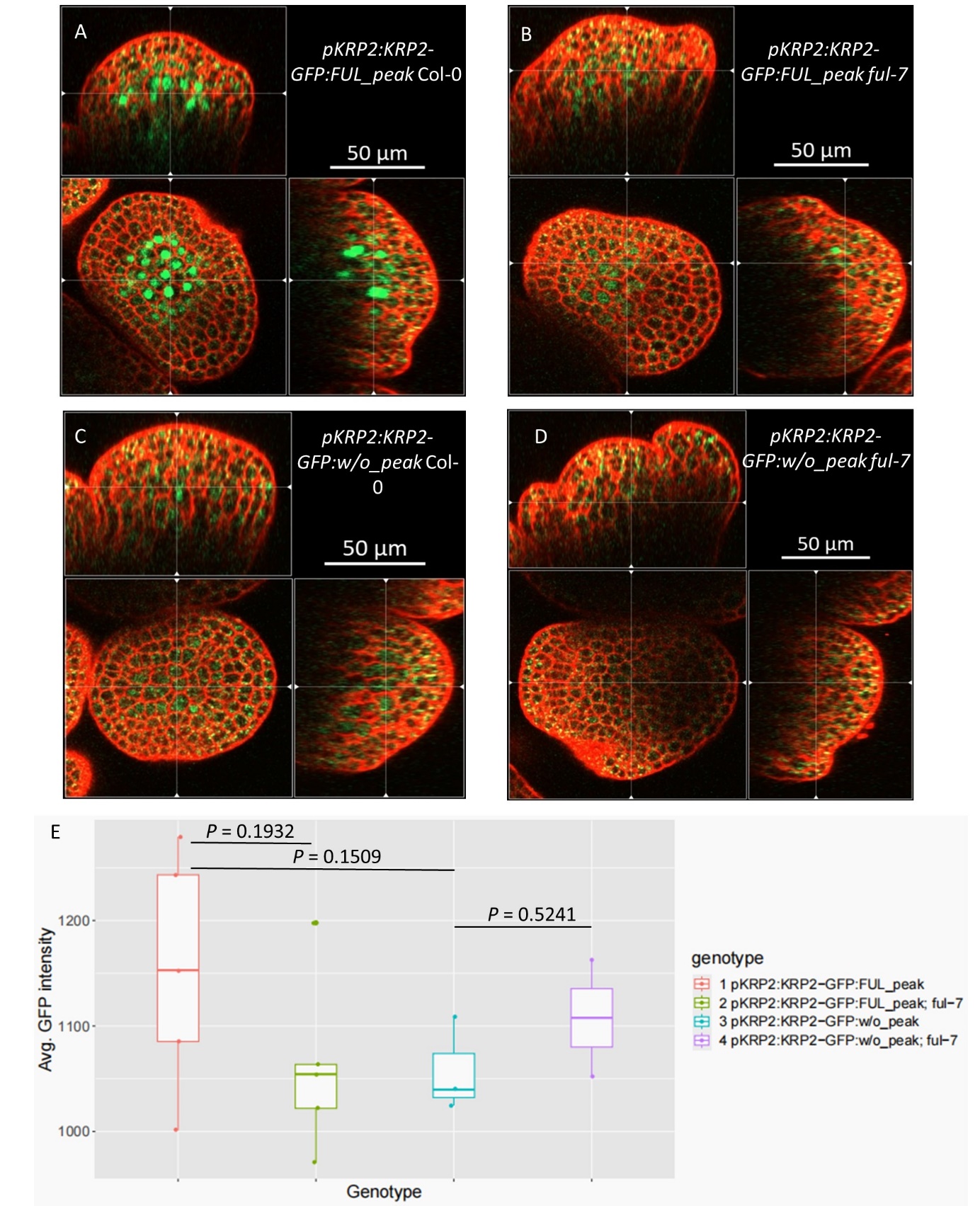
**

**Fig. S8. Confocal analysis of KRP2-GFP intensity in stage 3 flowers.** **A-D)** Confocal images of stage 3 flowers with ortho views. The genotypes are: **A)** *pKRP2:KRP2-GFP:FUL_peak* (N = 5), **B)** *pKRP2:KRP2-GFP:FUL_peak ful-7* (N = 5), **C)** *pKRP2:KRP2-GFP:w/o_peak* (N = 3), **D)** *pKRP2:KRP2-GFP:w/o_peak ful-7* (N = 2). The plants used for scanning are at a similar age with only 1-3 open flowers. **E)** Average GFP intensity of stage 3 flower meristem cells. Bilateral *t*-test was used to test differences.

**
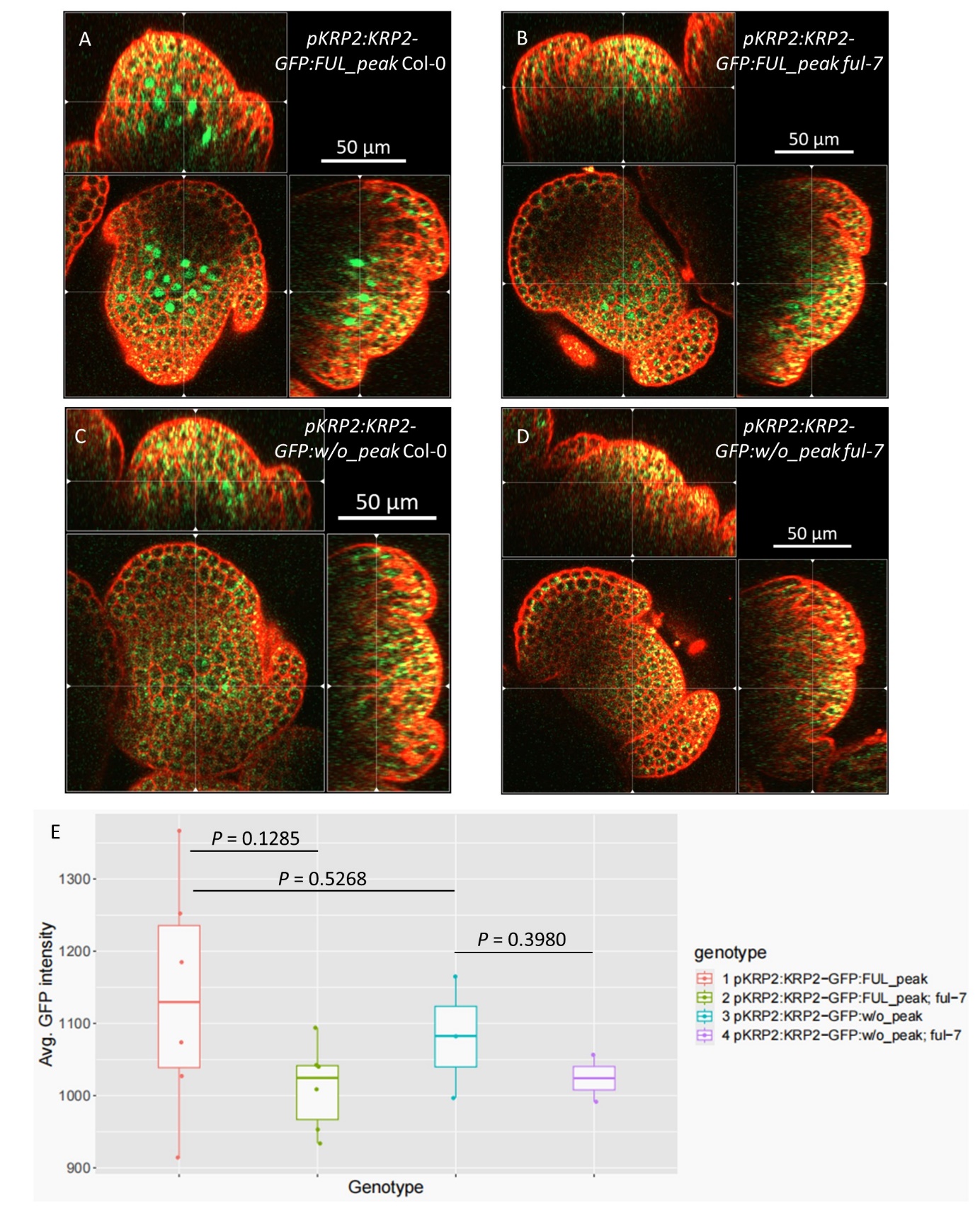
**

**Fig. S9. Confocal analysis of KRP2-GFP intensity in stage 4 flowers.** **A-D)** Confocal images of stage 4 flowers with ortho views. The genotypes are: **A)** *pKRP2:KRP2-GFP:FUL_peak* (N = 6), **B)** *pKRP2:KRP2-GFP:FUL_peak ful-7* (N = 6), **C)** *pKRP2:KRP2-GFP:w/o_peak* (N = 3), **D)** *pKRP2:KRP2-GFP:w/o_peak ful-7* (N = 2). The plants used for scanning are at a similar age with only 1-3 open flowers. **E)** Average GFP intensity of stage 4 flower cells. Bilateral *t*-test was used to test differences.


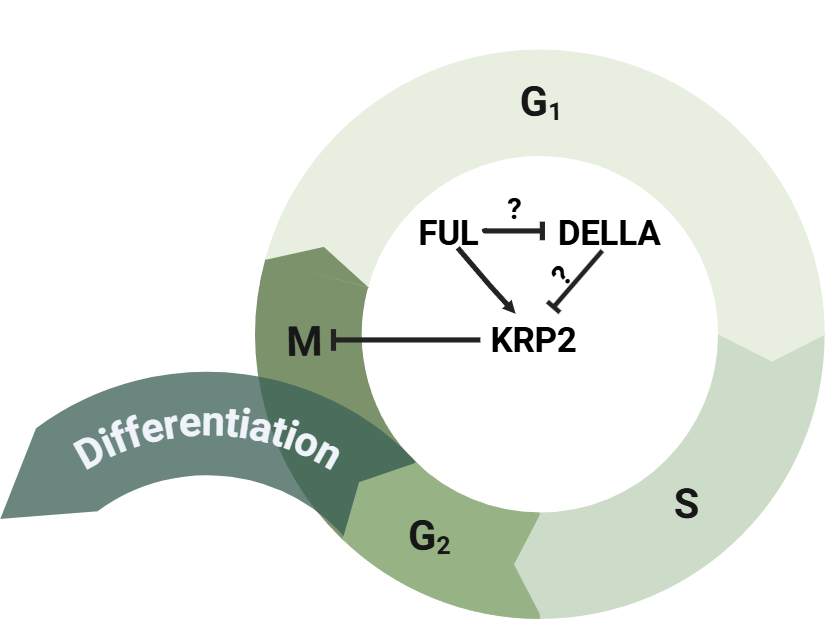


**Fig. S10. Summary of the FUL-KRP2 regulation.** FUL is able to bind a regulatory region downstream of *KRP2* and regulate *KRP2* expression as validated by *in vivo* and *in vitro* experiments in this study. KRP2 is reported to control G2-to-M transition (see Background section). Our FUL ChIP-seq data also shows that FUL could potentially regulate several DELLA genes (*RGA1* and *RGL2*). These DELLA proteins are involved in the regulation of *KRPs* expression (5).

**References:**

1. Yan W, Chen D, Schumacher J, Durantini D, Engelhorn J, Chen M, et al. Dynamic control of enhancer activity drives stage-specific gene expression during flower morphogenesis. Nat Commun. 2019 Apr 12;10(1):1705.

2. Yadav RK, Tavakkoli M, Xie M, Girke T, Reddy GV. A high-resolution gene expression map of the Arabidopsis shoot meristem stem cell niche. Dev Camb Engl. 2014 Jul;141(13):2735–44.

3. van Mourik H, Chen P, Smaczniak C, Boeren S, Kaufmann K, Bemer M, et al. Dual specificity and target gene selection by the MADS-domain protein FRUITFULL. Nat Plants. 2023 Mar;9(3):473–85.

4. Smaczniak C, Immink RGH, Muiño JM, Blanvillain R, Busscher M, Busscher-Lange J, et al. Characterization of MADS-domain transcription factor complexes in Arabidopsis flower development. Proc Natl Acad Sci. 2012 Jan 31;109(5):1560–5.

5. Serrano-Mislata A, Bencivenga S, Bush M, Schiessl K, Boden S, Sablowski R. DELLA genes restrict inflorescence meristem function independently of plant height. Nat Plants. 2017 Sep;3(9):749–54.
